# Dynamic thromboembolic left ventricular outflow tract obstruction after aggressive procoagulant treatment in hemorrhagic shock: a case report

**DOI:** 10.1186/s13256-021-02840-3

**Published:** 2021-05-18

**Authors:** Vladimir Skrypnikov, Christoph Rosenthal, Steffen Weber-Carstens, Mario Menk, Martin Russ

**Affiliations:** 1 Department of Anesthesiology and Intensive Care Medicine (CVK, CCM) Charité - Universitätsmedizin Berlin, Corporate Member of Freie Universität Berlin, Humboldt-Universität zu Berlin, and Berlin Institute of Health, Augustenburger Platz 1, 13357 Berlin, Germany; 2 Department of Anesthesiology, Intensive Care Medicine, Emergency Medicine and Pain Therapy, Vivantes Klinikum im Friedrichshain, Landsberger Allee 49, 10249 Berlin, Germany; 3 Department of Anesthesiology and Operative Intensive Care Medicine, Campus Virchow Klinikum Charité - Universitätsmedizin Berlin, Augustenburger Platz 1, 13353 Berlin, Germany

**Keywords:** Hemorrhagic shock, Dynamic LVOT obstruction, SAM phenomenon, Pro-coagulatory therapy, Thrombotic complication

## Abstract

**Background:**

In cases of hypertrophic obstructive cardiomyopathy (HOCM), the systolic anterior motion of the mitral valve apparatus results in an obstruction of the left ventricular outflow tract (LVOT), which is known as the SAM [systolic anterior motion] phenomenon. Hypothetically, a pathological obstruction of the LVOT of a different etiology would result in a comparable hemodynamic instability, which would be refractory to inotrope therapy, and may be detectable through echocardiography.

**Case presentation:**

We observed a severely impaired left ventricular function due to a combination of a thrombotic LVOT obstruction and distinctive mitral regurgitation in a 56-year-old Caucasian, female patient after massive transfusion with aggressive procoagulant therapy. Initially, the patient had to be resuscitated due to cardiac arrest after a long-distance flight. The resuscitation attempts in combination with lysis therapy due to suspected pulmonary artery embolism were initially successful but resulted in traumatic liver injury, hemorrhagic shock and subsequent acute respiratory distress syndrome (ARDS). Oxygenation was stabilized with veno-venous extracorporeal membrane oxygenation (ECMO), but the hemodynamic situation deteriorated further. Transesophageal echocardiography (TEE) showed a massive, dynamic LVOT obstruction. Two thrombi were attached to the anterior leaflet of the mitral valve, resulting in a predominantly systolic obstruction. Unfortunately, the patient died of multiple-organ failure despite another round of lysis therapy and escalation of the ECMO circuit to a veno-venoarterial cannulation for hemodynamic support.

**Conclusion:**

Massive transfusion with aggressive procoagulant therapy resulted in mitral valve leaflet thrombosis with dynamic, predominantly systolic LVOT obstruction, comparable to the SAM phenomenon. The pathology was only detectable with a TEE investigation.

**Supplementary Information:**

The online version contains supplementary material available at https://doi.org/10.1186/s13256-021-02840-3.

## Background

Dynamic obstruction of the left-ventricular outflow tract is a rare condition. The known common causes of left ventricular outflow tract (LVOT) obstruction include hypertrophic obstructive cardiomyopathy (HOCM), dehydration, sepsis, cardiac surgical treatment after valve repair, and Takotsubo cardiomyopathy [1].

In the case reported here, the LVOT obstruction was caused by thrombotic formations. The thrombotic structures situated at the mitral valve caused a severe systolic obstruction of the LVOT comparable to the so-called SAM [systolic anterior motion] phenomenon [2]. In this condition, the anterior leaflet (cusp) moves towards the LVOT and obstructs the outflow tract. This leads to the extension of the systolic ejection phase, a decrease in the ejection volume and increased pressure work for the left ventricle. Also, a posterior mitral insufficiency was caused by an insufficient coaptation of the mitral leaflets. This further impaired cardiac output and led to abrupt hypotension and a low cardiac output syndrome. The SAM phenomenon is known in patients with hypertrophic obstructive cardiomyopathy (HOCM) or in the condition of severe volume depletion [3–6]. The condition is a dynamic phenomenon and can—albeit rarely—be a cause of hypotension [7–9].

We present the case of a rare thrombus formation attached to the mitral valve under procoagulant therapy, massive transfusion and extracorporeal membrane oxygenation (ECMO) therapy despite continued anticoagulation, which resulted in a unique dynamic obstruction of the LVOT similar to a SAM phenomenon.

## Case presentation

**Fig. 1:**
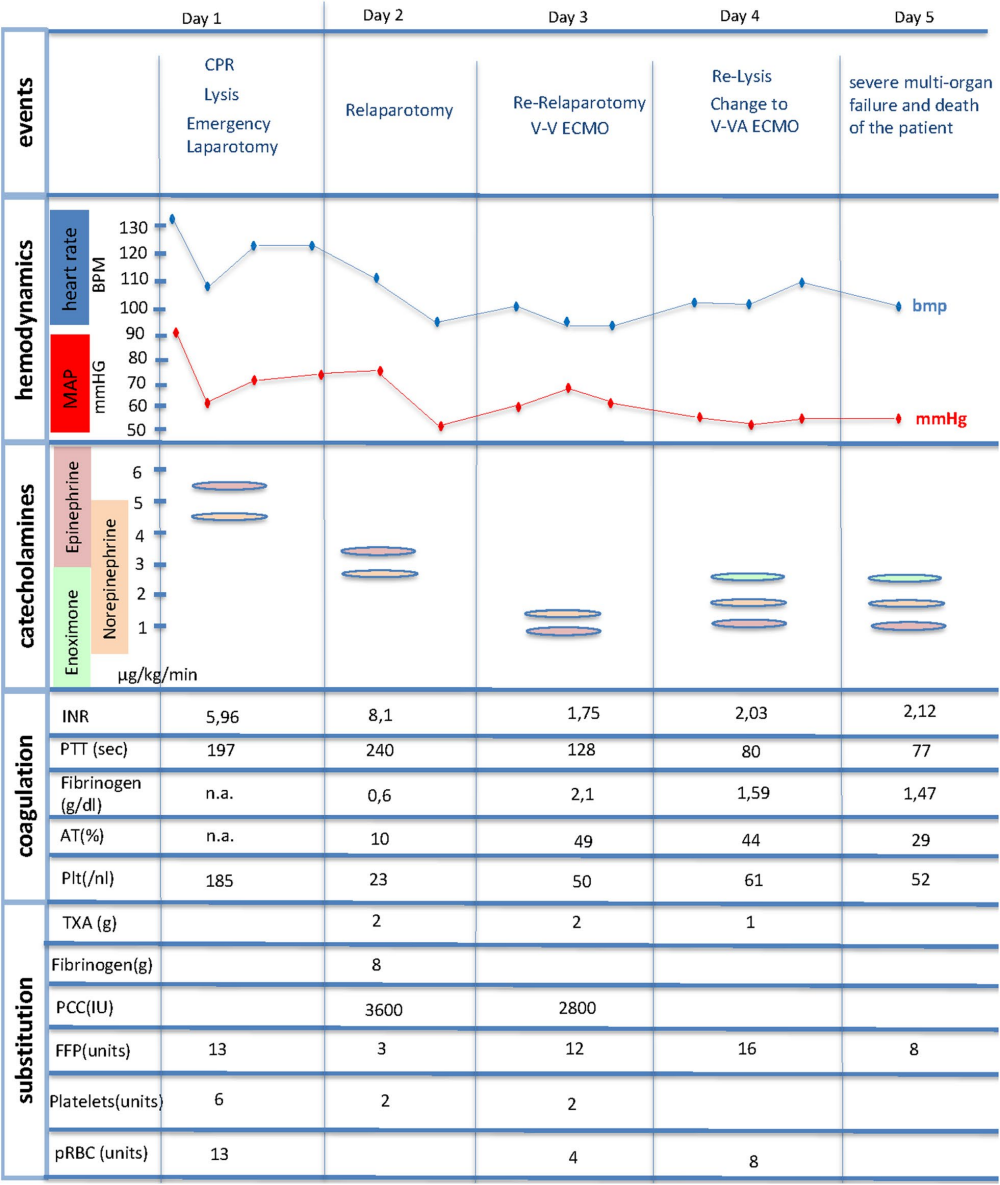
Schematic presentation of interventions, hemodynamics, catecholamine therapy, coagulation parameters and substituted clotting preparations over time. Interventions, hemodynamic data, laboratory results and all clotting preparations and blood products which were substituted or transfused during the course of treatment are shown. A representative catecholamine dosage is provided for each day of treatment. *CPR* cardiopulmonary resuscitation, *V-V ECMO* veno-venous extracorporeal membrane oxygenation, *V-VA ECMO* veno-venoarterial extracorporeal membrane oxygenation, *MAP* mean arterial pressure, *bpm* beats per minute, *INR* international normalized ratio of prothrombin time, *PTT* partial thromboplastin time, *AT* antithrombin concentration, *Plt* platelet concentration, *TXA* tranexamic acid, *PCC* prothrombin complex concentrate, *IU* international unit, *FFP* fresh-frozen plasma, *pRBC* packed red blood cells

A 56-year-old woman (Caucasian, married, two children) without preexisting conditions, who had previously performed moderate endurance runs about 2-3 times per week according to her relatives, suffered from acute cardiac arrest at home. Prior to cardiac arrest she had complained of pain in her calf after a long-distance flight. The first rhythm detected by the emergency service crew was pulseless electrical activity. During pre-clinic cardiopulmonary resuscitation, systemic lysis therapy (tenecteplase, 100 mg) was initiated under suspicion of pulmonary embolism. After the return of spontaneous circulation, the patient was transferred to an emergency department. At arrival at the resuscitation area, the patient presented with hemodynamic shock (mean arterial pressure, MAP of 92 mmHg, heart rate 133 beats/minute, epinephrine dosage of 5.5 µg/kg/minute, norepinephrine dosage of 4.5 µg/kg/minute), was intubated, mechanically ventilated and was unresponsive (Glasgow Coma Scale score of 3) without further sedation (the patient had received 15 mg midazolam, 0.5 mg fentanyl during the initial resuscitation). The first computed tomography (CT) scan did not reveal a pulmonary embolism. The treating team assumed that a pulmonary embolism, which was completely lysed, was the most likely explanation for the cardiac arrest based on the typical clinical presentation, case history and good response to lysis therapy. Shortly thereafter, the hemodynamic situation deteriorated again with requirement for massive doses of catecholamines, a pronounced drop in hemoglobin levels and the need for massive transfusion. The CT scan showed a hemoperitoneum due to a traumatic rupture of the liver after resuscitation. An emergency laparotomy was performed, the liver was sutured and the abdomen was packed. During surgery, a prolonged, massive transfusion of packed red blood cells, fresh-frozen plasma and thrombocyte concentrates was necessary. Further, coagulation factors including fibrinogen, antithrombin (AT) and four-factor prothrombin complex concentrate (4F-PCC) were administered (Fig. 1).

On the following day, the patient developed an abdominal compartment syndrome with the onset of acute liver failure and the need for a re-laparotomy. She also developed progressive lung failure with highly elevated parameters of mechanical ventilation and poor oxygenation. Therefore, she was transferred to our ECMO center, where veno-venous (V-V) ECMO was implemented.

**Fig. 2:**
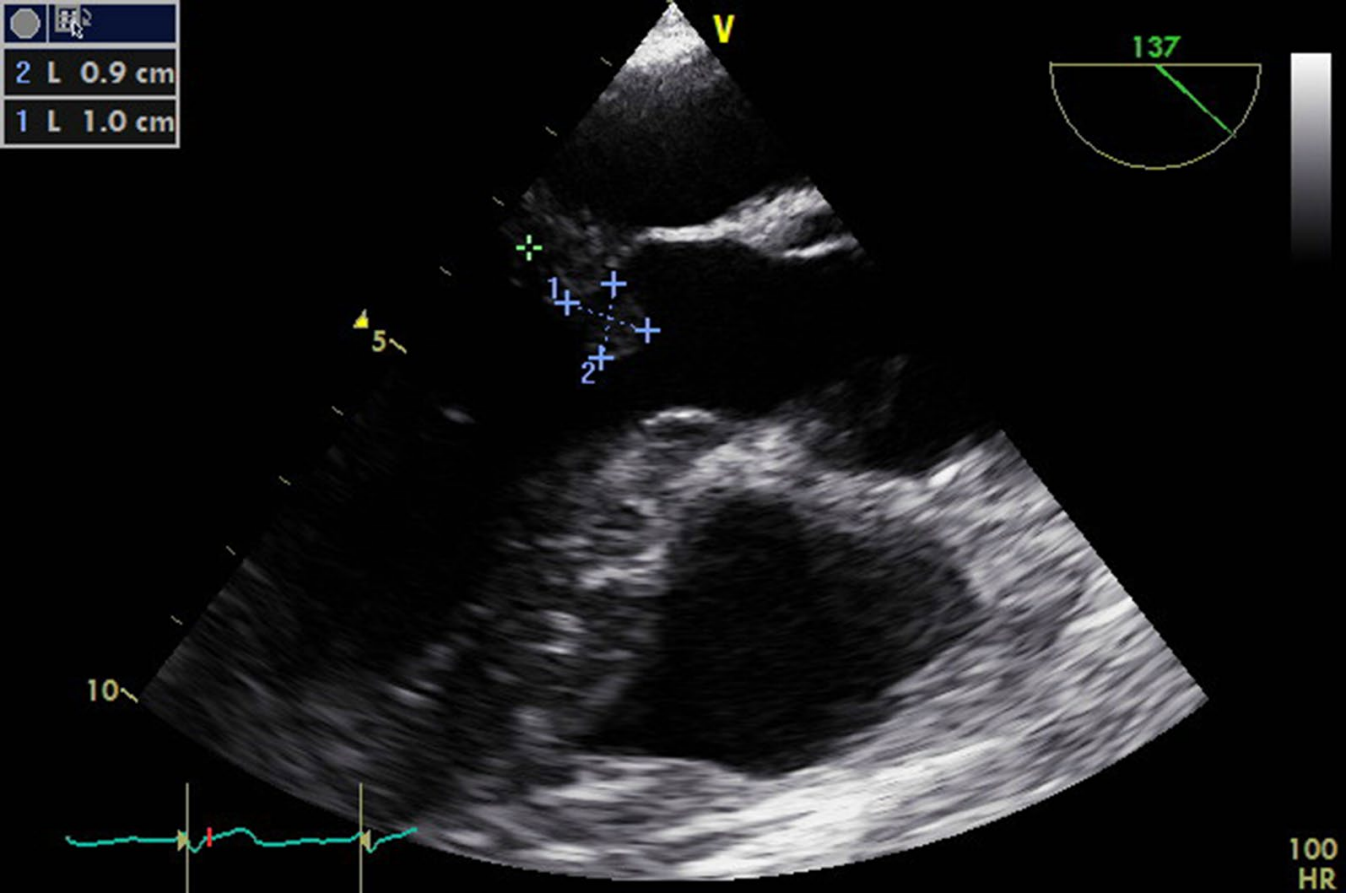
Midesophageal longaxis view (137°): showing obstructive mass on anterior mitral leaflet ~ 0.9 cm x 1.0 cm in left ventricular outflow tract during systole. The hypodense structure at the mitral valve causes obstruction of the left ventricular outflow tract and significantly reduces left-ventricular ejection volume and cardiac output

Transesophageal echocardiography (TEE) revealed severe right-heart stress (severe right ventricular dilatation, RV/LV > 2, decreased septal wall motion, severe tricuspid regurgitation, volume depletion of left ventricle) and beginning right-heart failure. Consequently, V-V ECMO was extended to venous-venoarterial (V-VA) cannulation with a 23 French, 38-cm-long drainage cannula (right femoral vein), 19 French 50-cm-long, venous return cannula (left femoral vein) and 15 French, 15-cm-long arterial return cannula (left femoral artery), that is, with a continuous cardiopulmonary bypass flow. Anticoagulation with an activated partial thromboplastin time (aPTT) above 60 seconds was performed with unfractionated heparin. Further, echocardiography revealed two pronounced, hyperdense thrombotic structures located directly at both leaflets of the mitral valve. The thrombus located at the anterior leaflet measured 0.9 × 1.0 cm and caused an obstruction of the LVOT during systole (Fig. 2, Additional file 1: Video S1, Additional file 3: Video S3).

**Fig. 3:**
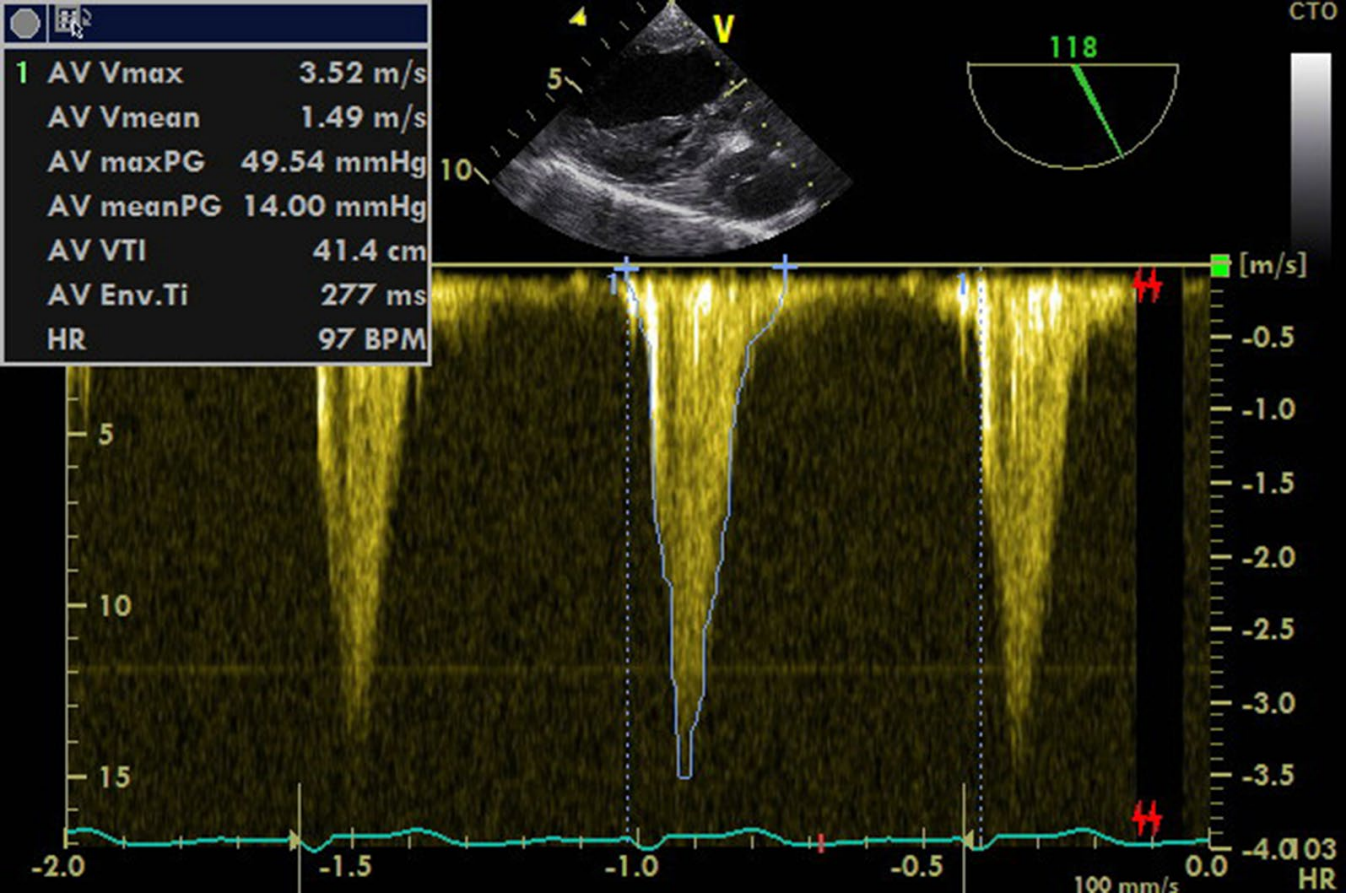
Transgastric long-axis view at 118°. Continuous-wave Doppler shows 3.5 m/second velocity across left ventricular outflow tract with indicating moderate obstruction

**Fig. 4.**
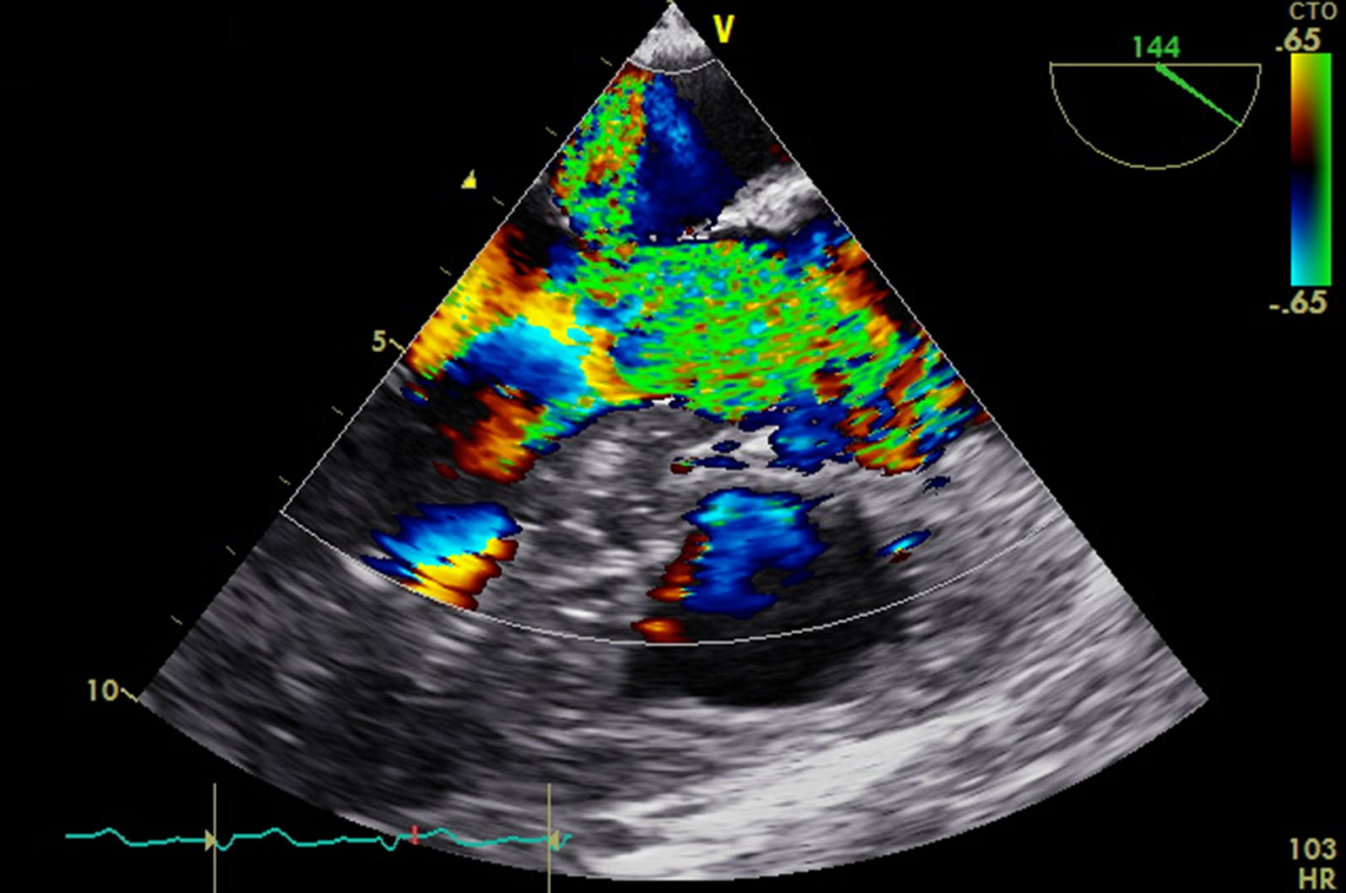
Mid-esophageal long-axis view at 144° showing mitral jet during systole, showing moderate mitral insufficiency

**Fig. 5.**
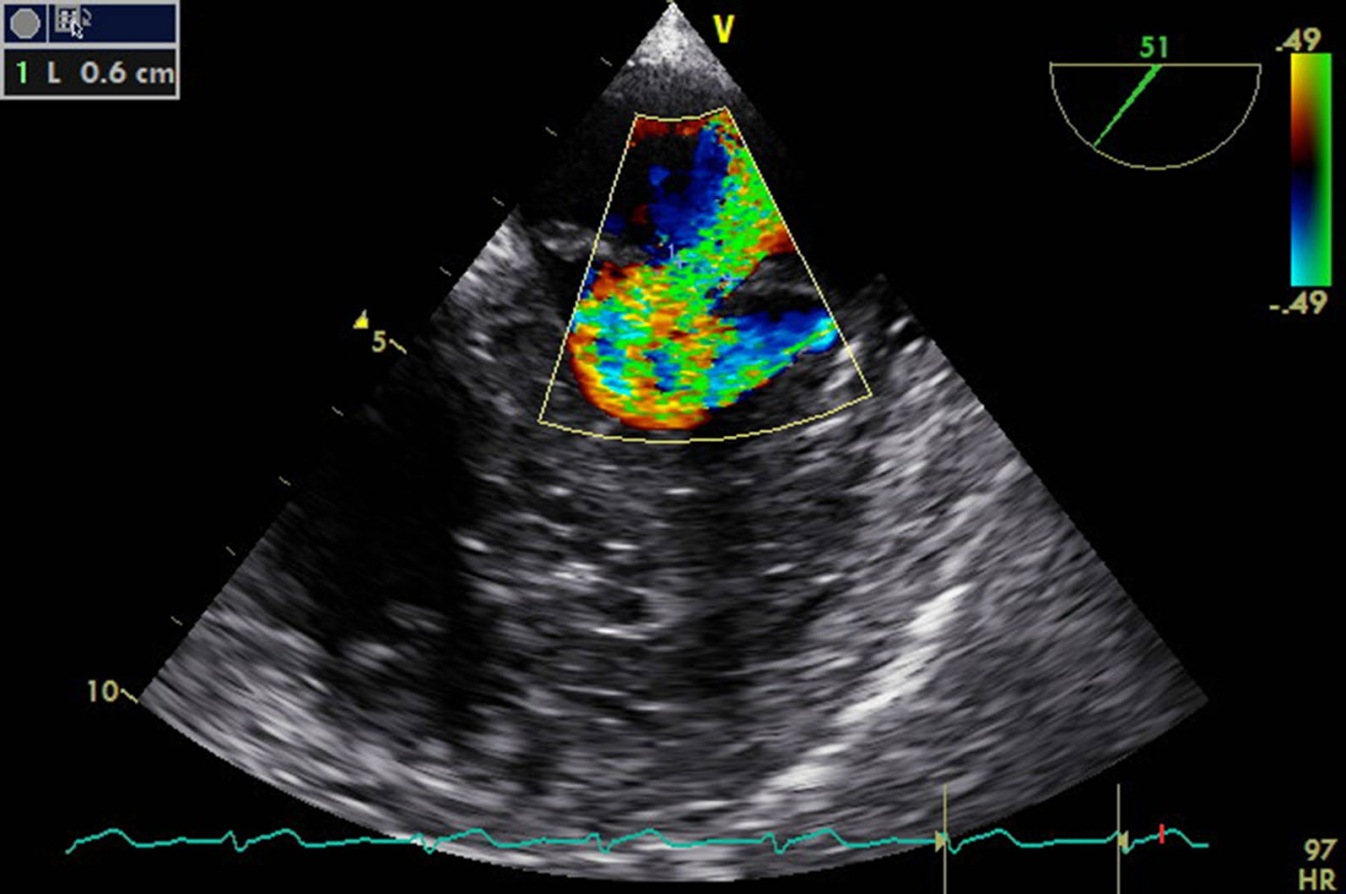
Mid-esophageal view at 50° showing moderate mitral valve regurgitation with vena contracta ~ 0.6 cm

*V*_max_ in the LVOT was 3.52 m/second (*P*_max_ of 50 mmHg) (Fig. 3). Also, a moderate mitral insufficiency with a vena contracta of 0.6 cm was found (Figs. 4, 5, Additional file 2: Video S2). Of note, both the thrombus and the tip of the anterior mitral leaflet (AMVL, which was enclosed by the thrombus) obstructed the LVOT in a dynamic fashion (Additional file 3: Video S3), which was aggravated during systole and may be comparable to the SAM phenomenon in its hemodynamic consequence. Furthermore, the mitral insufficiency was an indirect indicator of a SAM-like pathology if considered with respect to the systolic motion of the thrombus and the tip of the AMVL.

Clinically, the patient remained highly unstable, demanding high dosages of catecholamines despite V-VA ECMO support. A new *ultima ratio* CT scan to rule out other causes of hemodynamic instability showed a previously undescribed central, right pulmonary embolism with almost complete obstruction of the right upper pulmonary artery, as well as numerous small thrombi in several left and right lung segments. In this situation with thromboembolic obstruction of both LVOT and pulmonary arteries, another systemic lysis treatment (alteplase, 30 mg) was initiated as *ultima ratio* therapy. Echocardiography performed shortly after lysis revealed no remaining mitral thrombi and an unobstructed, free LVOT. In parallel, the catecholamine dosages were decreased substantially, but the patient could not be weaned completely at any point.

Nevertheless, the patient developed progressive acute liver failure with increasing lactic acidosis and high demand for glucose substitution, pulmonary failure without improvement in oxygenation or decarboxylation (ECMO gas flow could not be reduced) and renal failure. Finally, the patient died from severe multiple-organ failure despite V-VA ECMO therapy. Postmortem examination revealed the disseminated formation of intravascular thrombi, for instance in the *vena femoralis*, pulmonary arteries, *vena porta hepatis* and *vena lienalis*.

## Discussion and conclusions

To the best of our knowledge, this is the first report of a dynamic LVOT obstruction as a direct consequence of procoagulant therapy and the subsequent formation of a massive thrombus, which was attached to the anterior leaflet of the mitral valve.

The condition of hemorrhagic shock is complex, and the imbalance in the coagulation processes can substantially increase the risk of intravascular thrombosis [4]. A differentiated and well-balanced treatment applying both pro- and anti-coagulatory factors (such as heparin, AT) has been shown to be a relevant part of therapy for severe bleeding [5]. In our case, AT levels were far below 20% at intensive care unit (ICU) admission. Thus, we speculate that the excessive substitution of pro-coagulatory factors and the subsequent imbalance of the coagulation system was—at least in part—causative for the thrombotic complications seen in our patient. 4F-PCCs carry an increased prothrombotic potential. In trauma patients, Schöchl *et al*. described an increased endogenous thrombin potential after administration of 4F-PCC [10]. The benefit of PCC compared to fresh-frozen plasma with its decreased volume load to the patient (especially in pulmonary and cardiac failure) has to be weighed against the increased prothrombotic risk. It would have been ideal to use viscoelastic tests combined with a point-of-care testing (POCT)-based coagulation management algorithm for monitoring of coagulation status and for goal directed coagulation management in this highly dynamic situation to avoid procoagulant overstimulation. Unfortunately, at the time of treatment of this patient, we did not have a well-established POCT-based coagulation management algorithm.

A bacterial endocarditis has to be considered as a differential diagnosis of structures at the mitral valve. However, in the case reported here, no signs of ongoing or florid infections were seen before the critical incident. Also, postmortem examination revealed no evidence for a pre-existing or new endocarditis.

Of importance, low flow situations on ECMO, especially V-A ECMO, such as increased afterload caused by the jet from the return cannula, can cause or aggravate LVOT thrombi. However, in the case presented here, the mitral valve thrombus was detected before the ECMO therapy was escalated to V-VA ECMO. The ECMO circuit (pump, oxygenator, ECMO lines) did not show any thrombus formation. Thus, we think that the combination of massive transfusion with aggressive procoagulant therapy was the major cause of the mitral valve leaflet thrombosis. Nevertheless, phases of reduced flow during hemodynamic decompensation in our patient as well as LV underfilling (during bleeding, surgery and pulmonary embolism) might have contributed to the thrombus formation which was detected with TEE.

Of note, the case confirms the essential role of echocardiography in the diagnosis and treatment of critically ill cardiovascular unstable patients in the ICU [11]. None of the common methods of hemodynamic monitoring such as invasive blood pressure measurement, pulse index contour cardiac output (PiCCO) or pulmonary artery catheterization provides sufficient information on intracardiac pathologies. Echocardiography with evaluation of the LVOT was essential for diagnosis and treatment of the condition in the present case. Even if the thrombi could have been visualized with a CT scan, only the moving real-time images of an ultrasound investigation displayed the systolic obstruction of the LVOT. In our opinion, echocardiography should be regarded as the diagnostic procedure of choice in cardiovascular unstable patients on the ICU whenever possible.

## Supplementary Information

**Additional file 1: Video S1.** Midesophageal longaxis view at 137°: obstructive mass on anterior mitral leaflet in left ventricular outflow tract during systole.

**Additional file 2: Video S2.** Midesophageal longaxis view (137°): obstructive mass anterior mitral leaflet in left ventricular outflow tract during systole (slow motion).

**Additional file 3: Video S3.** Mid-esophageal long-axis view (137°): obstructive mass on mitral valve (AMVL) in LVOT during systole (slow motion).

## Data Availability

All data generated or analyzed during this study are included in this published article and its additional information files.

## Abbreviations

AMVL: Anterior mitral leaflet
ARDS: Acute respiratory distress syndrome
AT: Antithrombin
CT: Computed tomography
ECMO: Extracorporeal membrane oxygenation
HOCM: Hypertrophic obstructive cardiomyopathy
LVOT: Left ventricular outflow tract
PiCCO: Pulse index contour cardiac output
POCT: Point-of-care testing
SAM: Systolic anterior motion
TEE: Transesophageal echocardiography
V-V: Veno-venous
V-VA: Venous-venoarterial
4F-PCC: Four-factor prothrombin complex concentrate
